# Review: Current trends, challenges, and success stories in adjuvant research

**DOI:** 10.3389/fimmu.2023.1105655

**Published:** 2023-01-19

**Authors:** Kentner L. Singleton, Ari Joffe, Wolfgang W. Leitner

**Affiliations:** Division of Allergy Immunology and Transplantation, National Institute of Allergy and Infectious Diseases, National Institutes of Health, Bethesda, MD, United States

**Keywords:** adjuvant discovery, adjuvant formulation, immune potentiator, innate immune receptor, pathogen-associated immunomodulator, reactogenicity

## Abstract

Vaccine adjuvant research is being fueled and driven by progress in the field of innate immunity that has significantly advanced in the past two decades with the discovery of countless innate immune receptors and innate immune pathways. Receptors for pathogen-associated molecules (PAMPs) or host-derived, danger-associated molecules (DAMPs), as well as molecules in the signaling pathways used by such receptors, are a rich source of potential targets for agonists that enable the tuning of innate immune responses in an unprecedented manner. Targeted modulation of immune responses is achieved not only through the choice of immunostimulator – or select combinations of adjuvants – but also through formulation and systematic modifications of the chemical structure of immunostimulatory molecules. The use of medium and high-throughput screening methods for finding immunostimulators has further accelerated the identification of promising novel adjuvants. However, despite the progress that has been made in finding new adjuvants through systematic screening campaigns, the process is far from perfect. A major bottleneck that significantly slows the process of turning confirmed or putative innate immune receptor agonists into vaccine adjuvants continues to be the lack of defined *in vitro* correlates of *in vivo* adjuvanticity. This brief review discusses recent developments, exciting trends, and notable successes in the adjuvant research field, albeit acknowledging challenges and areas for improvement.

## 1 Introduction

With the move from inactivated pathogen-based vaccines that deliver endogenous immune stimulatory signals to subunit vaccines, the demand for potent and safe adjuvants has increased. While “clean” recombinant proteins depend on strong exogenous adjuvants, even traditional, whole-pathogen vaccines can benefit from the addition of adjuvants. An excellent, recent example is the development of adjuvanted inactivated pathogen (SARS-CoV-2)-based COVID vaccines such as Covaxin or CoronaVac. In addition, an MF59-adjuvanted, inactivated influenza vaccine (Fluad) (1) is licensed in more than 20 countries worldwide and can address poor responses to influenza vaccines in the elderly, which are due to immunosenescence.

The slow approval of vaccines that contain novel adjuvants may have made the field of adjuvant research appear rather stagnant to outsiders. However, the preclinical adjuvant development pipeline is quite robust, and received an unprecedented - and unexpected - boost by the COVID pandemic that also benefits efforts to develop new or better vaccines for other infectious diseases. This article only focuses on vaccine adjuvants that target innate immune pathways and receptors, but it is important to acknowledge immunostimulators that directly target lymphocytes, such as the Complement fragment C3d (reviewed in (2)).

## 2 New trends in adjuvant research

### 2.1 Synthetic and small molecule adjuvants

The way immunostimulators are identified has dramatically changed over time. Adjuvants have traditionally been the result of chance discoveries, whether they are non-naturally occurring formulations and compounds (*e.g.*, aluminum salts (“alum”), emulsions such as Freund’s Adjuvant) that have unexpected adjuvant activity, or pathogen-derived immunostimulators (*e.g.*, proteins such as double-mutant heat labile toxin (dmLT^1^) or certain DNA sequences (CpG oligodeoxynucleotides (ODNs)). More recently, adjuvant discovery has become a highly targeted effort whereby immunostimulators are identified using screening campaigns, often targeting a specific innate immune receptor or pathway. High-throughput screening approaches have long been used for drug discovery and have revolutionized adjuvant discovery, not only by accelerating the speed of discovery, but also by shifting the focus from canonical innate immune receptor agonists to small molecule immunopotentiators (SMIPs). An excellent example of this transition is the field of TLR4 agonist research: Monophoshoryl lipid A (MPL (3)), first developed in the 1970s and currently used in Shingrix and Mosquirix, was created through chemical modifications of lipopolysaccharide from *Salmonella minnesota*. At the time, TLR4 had not yet been identified as the receptor for lipopolysaccharide (AKA endotoxin), which was known to be a strong inducer of inflammation. Subsequent generations of what we now know to be TLR4 agonists (such as GLA/PHAD^2^, INI-2002^3^, or CRX-601 (4)) are synthetic derivatives of MPL from which non-essential components have been removed. Minor modifications of the TLR4 agonists’ structures are able to significantly alter the resulting receptor signals (5). The latest generation of TLR4 agonists (*e.g*., 2B182c^4^ or neoseptin (6)) are small molecules that do not share structural similarities with the canonical receptor agonists. Small molecules often have multiple advantages compared to natural products, such as simpler – and cheaper – manufacturing, high stability, and more options for formulation.

### 2.2 From *in vivo* to (high throughput) *in vitro* adjuvant screening

**Figure 1:**
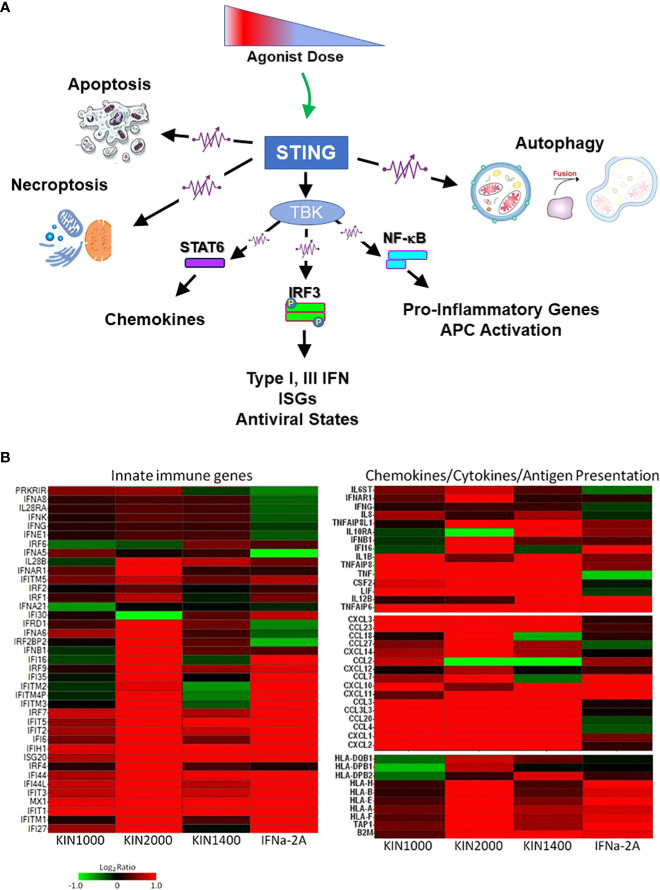
*Innate immune receptors as tunable, not binary switches*. **(A)** Engaging innate immune receptors such as the intracellular STING (Stimulator of Interferon Genes), a sensor of cytosolic pathogen derived or self-DNA, triggers a spectrum of downstream responses that quantitatively change depending on the dose of the receptor agonist. This supports the concept of innate immune receptors functioning like rheostats (or “dimmers”) rather than switches. **(B)** In addition to *quantitative* changes in the response pattern, different agonists (including small changes in the chemical structure of an agonist) can alter the receptor’s response pattern *qualitatively* as shown here for three small-molecule RIG-I agonists (first described in (7)). The gene expression-profile induced by IFNα-2A is included as a reference. RIG-I (Retinoic acid-inducible Gene-I, RLR-1) is a cytosolic receptor for specific motifs associated with viral RNA and used here as an example for how different synthetic agonists uniquely affect the downstream expression pattern of innate immune, chemokine, and cytokine genes or genes associated with antigen expression. This phenomenon had first been described for TLR4 that signals through two distinct signaling pathways (MyD88-dependent, and MyD88-independent/TRIF-dependent) that could be triggered differentially. However, it also applies to receptors that only utilize one signaling pathway (such as RIG-I, STING, or all other TLRs). The observation suggests that even the rheostat-model of innate immune receptors may be overly simplistic, and a more accurate electronic comparator might be multi-color LED dimmer switches that not only change the intensity of the signal (LED brightness/level of gene expression), but also the quality of the signal (LED color/gene expression pattern). Data provided by M.Gale and RC Ireton, University of Washington, Seattle WA.

“Classic” adjuvants were identified as immunostimulators based on their – in some cases unexpected – activity *in vivo*, such as various food ingredients. These include tapioca, starch, and lecithin that were screened for adjuvant activity in horses by Gaston Ramon, or aluminum salts that were used in an attempt to improve diphtheria vaccines by Alexander Glenny, both a century ago. Since then, reporter cell lines have facilitated high-throughput screening approaches for adjuvant-associated cellular activity *in vitro*. While such approaches can significantly simplify and speed up the process of identifying novel adjuvant candidates, moving from animals to cells in culture comes with an important caveat: a lack of known *in vitro* correlates of *in vivo* adjuvanticity. The use of reporter cells that indicate signaling through a particular innate immune receptor or signaling pathway has helped find many novel immune stimulators that are currently in the adjuvant development pipeline. However, it is important to keep in mind that many strong hits from such *in vitro* screens that rely on a reporter gene or the release of a specific cytokine, have shown little to no activity *in vivo*. Such setbacks serve as a reminder that many, if not all, innate immune receptors demonstrate three important properties: a) they act like biological rheostats rather than binary switches (**Figure 1**), with a “tunable” response (this phenomenon was first shown with TLR4 agonists that preferentially signal through either the TRIF or MyD88 pathway, subsequently altering the quality of the resulting immune response (8)); b) the result of stimulating such receptors is the induction or upregulation of a wide spectrum of molecules, including cytokines and cell-surface markers; and c) the signals – and, thus, the response-patterns - that a specific PRR agonist induces in different cell types is not necessarily the same (as discussed in more detail in the next section). Therefore, it is essential for the adjuvant research field to move beyond single-parameter readouts of adjuvant-induced responses and towards “unbiased” multi-parameter readouts (9), paired with computational data integration and machine learning (10). It can - and indeed should - be expected that an initial, broad profiling of responses induced by an immunostimulator will yield many parameters, such as certain cytokines, that do not correlate with adjuvanticity. It is also highly likely that no single parameter will be identified that can predict an immunostimulator’s ability to provide good *in vivo* adjuvanticity (10). A panel of immune parameters (including cytokines and the cells that produce them, chemokines, antibody isotypes and epitope specificity, markers on lymphocyte subsets, functional activities such as opsonophagocytosis (11)) that correlate with adjuvanticity can instead be used to establish a compound’s immunological signature or “fingerprint” (12). Such an adjuvant fingerprint will not only assist in rational adjuvant selection, but also bring us closer to the establishment of the so-far elusive *in vitro* correlates of adjuvanticity (an effort supported by the NIAID through its Adjuvant Comparison Program^5^). At the same time, the extensive profiling of immune responses by an adjuvanted vaccine, paired with efficacy data from controlled infection studies, will help with the identification of correlates of protection by vaccines. Subunit and killed-pathogen vaccines have created an expectation that vaccines must induce (neutralizing) antibodies in order to pass regulatory muster. Unfortunately, this somewhat artificial criterion slows the development – and approval – of vaccines that induce other effector mechanisms, such as effector T cells (for example, in case of a fungal vaccine (13)), tissue-resident memory T cells (for example, in case of Tuberculosis (14)), or antibodies that – counter-intuitively – inhibit phagocytosis of the pathogen (as in the case of malaria (15)).

For vaccine developers, the heterogeneity of the response induced by different immunostimulators within the same class represents both challenges and opportunities. Even closely related pattern recognition receptors (PRR) ligands, such as CpG ODNs that only differ in a single nucleotide within the CpG flanking region, or imidazoquinolines (IMDQs) with slightly different side chains outside the core structure, can induce significantly different immune responses. Therefore, it is impossible to judge the usefulness of a particular class of adjuvants for a specific vaccine based on the experience with a single representative from that class. While this necessitates conducting more extensive comparison studies of candidate adjuvants, it also increases the panel of available adjuvants that might be the best fit for a vaccine.

### 2.3 From *in vitro* to *in silico* adjuvant screening

The recognition that small molecules can mimic the binding of large pathogen-associated molecules to innate immune receptors has also opened the door for *in silico* modeling of their binding to receptors whose crystal structure is known. *In silico* docking studies that predict the binding strength of a molecule to an innate immune receptor have been used to pre-select molecules for a subsequent (more targeted) *in vitro* screen. In addition, they have been employed for predicting potential agonists before the physical molecule is even synthesized. The concept has enormous appeal since it can increase the throughput rate of an adjuvant screen to levels that cannot be achieved in a physical (*in vitro*) high-throughput screen. Not only some novel adjuvants are emerging from this approach (such as the above-mentioned TLR4 agonist 2B182c^6^), but also well-characterized drugs that are represented in compound libraries have been found to have unexpected adjuvant activity (such as the anti-fungal Amphotericin B (16)), which opens the door for repurposing. Nevertheless, the technology has not lived up to the high expectations quite yet. One major reason for the failure of many *in silico* hits to have any adjuvant activity *in vivo*, or even *in vitro*, is that binding to an innate immune receptor, even within the ligand binding site, does not necessarily translate into receptor activation. Some receptors require cross-linking in addition to receptor binding, which may be accomplished by tethering two copies of the potential agonist with a chemical linker to force receptor cross-linking. A remarkable success story of *in silico* adjuvant screening, albeit not of a small molecule adjuvant, is the discovery of novel CpG ODN sequences. In the absence of a full crystal structure of the receptor, a hypothetical structure of TLR9 was used for the *in silico* high-throughput screening campaign (17). This approach yielded CpG55.2, a multi-species active TLR9 adjuvant that acts synergistically with the carbohydrate Advax^7^ in Advax/CpG55.2^8^, that is used in the COVID vaccine Spikogen/Covax-19 (18).

### 2.4 From reporter cell lines to primary cells

Reporter cells that express select innate immune receptors and reporter gene(s) have become the workhorse of adjuvant discovery efforts. They are amenable to the high-throughput format, can reliably and reproducibly identify agonists of specific immune receptors, and are significantly less expensive to use than cytokine measurement-assays. However, just as *in silico*-predicted binding to an innate immune receptor does not necessarily predict activation of the receptor (as discussed above), even successful signaling from the receptor does not necessarily translate into adjuvant activity *in vivo*. Using innate immune cell lines such as the human monocytic THP-1 line instead of receptor transfectants for the identification of new immunostimulators can significantly improve the predictive value of the screen, as well as widen the repertoire of adjuvant targets. Specific innate immune cell lines can also be employed to identify ‘unconventional’ types of adjuvants, such as modulators of the Type I IFN signal by TLR agonists (19). The value of cell lines such as THP-1 (human) or DC2.4 and IC-21 (mouse) lies in the assumption that the same relevant innate immune signaling receptors and components are present as in primary cells, even if not necessarily at the same level. However, it has been shown in different models that THP-1 cells respond quite differently to stimuli than monocyte-derived macrophages (20), which led Tedesco et al. to conclude that “THP-1 should be regarded as a simplified model of human macrophages when investigating relatively straightforward biological processes, such as polarization and its functional implications, but not as an alternative source in more comprehensive immunopharmacology and drug screening programs” (21). It is, thus, not surprising that in high-throughput screening campaigns designed to identify novel adjuvants, many compounds that stimulate THP-1 cells are not, or only poor, activators of primary human lymphocytes, and vice-versa (personal communication: D. Dowling, BCH, Boston, MA). While one of the stated advantages of cell lines is the elimination of donor-to-donor variations associated with primary cells, THP-1 cell lines that are used in different labs have drifted sufficiently in terms of receptor expression and functionality (personal communication: E. Bergmann-Leitner, WRAIR, Silver Spring, MD). This makes it difficult to compare results from different labs. Finally, further complicating the experimental setup for identifying immunostimulators *in vitro* are the reported differential effects that sera from donors of different ages have on how lymphocytes respond to stimuli. Few specific factors responsible for this phenomenon have been identified yet, a prominent example being adenosine in newborn serum that interferes with Th1-polarizing signaling pathways (22). Other age-dependent differences include baseline serum cytokine levels (23) and serum metabolite profiles (24). These findings underscores the need to use autologous serum for lymphocyte cultures. They also contributes to the concept that innate immune stimulators engage a rheostat (as described above and **Figure 1**) rather than flipping a switch, with serum factors contributing to the modulation of the signal. The use of primary human lymphocytes and homologous serum undoubtedly complicates the design of screening campaigns for novel adjuvants, for example, by increasing the need for testing the same compounds in cells from multiple donors to account for donor-to-donor variations. However, it also opens the door for the discovery of a) adjuvants that work across the age-spectrum, b) adjuvants that may be particularly suitable for specific populations (25), and c) factors (such as serum components) and mechanisms responsible for reduced responsiveness to vaccines in certain populations.

Organoid cultures represent an exciting new tool for adjuvant research. By replicating or preserving the cellular - and possibly structural - integrity of lymphoid tissues, organoid cultures have the potential to model the complex interactions between immune cell-subsets and, thus, the induction of immune responses much more reliably than conventional cell cultures. A commonly used secondary lymphatic tissue used for such studies is tonsils. Such organoid cultures have already been employed for evaluating human immune responses to different types of vaccines as well as adjuvants (26). While organoid cultures are already an established tool for studying human (viral) pathogens (reviewed in (27)), their use for evaluating and selecting vaccine adjuvants is still at a very early stage.

### 2.5 Combination adjuvants: 1 + 1 ≠ 2

Following the trend in the field of vaccine research and the focus on individual pathogen-derived antigens, adjuvant research has also moved towards the development of agonists for single PRRs that trigger a single, defined receptor or pathway. From a manufacturing and a regulatory standpoint, this approach is highly attractive and high-profile examples of very successful single-antigen vaccines already exist (such as Shingrix, Heplisav). However, the approach has either failed when applied to more complex pathogens or has resulted in decreasing efficacy when targeting a single protein that is either variable (such as malaria blood stage antigens) or subject to mutations (such as the SARS-CoV-2 S protein). Similarly, infections that result in the induction of protective immunity trigger multiple innate immune pathways. This also applies to some vaccines, such as the Yellow Fever Vaccine that owes its ability to induce long-lasting protective immunity to the stimulation of multiple TLRs (28). The adjuvant field has responded to this challenge with the development of combination adjuvants. While conceptually very straight-forward, determining promising combinations requires not only the laborious identification of the optimal ratio of adjuvant partners, but also the careful characterization of which combination provides additive or truly synergistic enhancement of immune responses without simultaneously increasing the reactogenicity of the formulation. Some combinations may not be compatible and even result in antagonism (29). However, the approach holds enormous potential since it dramatically expands the availability of formulations that can be used to optimize a vaccine’s efficacy, while also potentially reducing reactogenicity by requiring lower doses of individual adjuvant components. Alum has traditionally been used as a stand-alone adjuvant for a wide variety of vaccines and calls to replace it with better, more ‘modern’ adjuvants have been around for many years. However, the COVID pandemic has highlighted the usefulness of Alum as a co-adjuvant in formulations where it serves primarily as a delivery platform that targets both antigen and co-adjuvant to the draining lymph node. An example of this approach is Alhydroxiquim-II^9^, the first TLR7/8 agonist in an approved infectious disease vaccine (Covaxin), and the first TLR agonist in a vaccine given to small children as young as 2 years old. While TLR7/8 agonists had previously been shunned for their reactogenicity, Alhydroxiquim-II has an exceptional safety profile since the potent TLR agonist is targeted to the draining lymph node, thus avoiding injection site - or systemic - reactogenicity. Another example is Advax/CpG55.2^8^ (described above), a novel combination adjuvant used in an approved COVID vaccine (Covax-19/Spikogen). In this combination, the carrier and co-adjuvant is crystalline carbohydrate (Advax^7^) rather than Alum. The synergy between the two adjuvant components allows dose sparing of the TLR9 agonist, which is further reduced by the anti-inflammatory nature of Advax. Additional alternatives to Alum as a carrier and co-adjuvant continue to come online, and a notable example is Microcrystalline Tyrosine (MCT^10^) that has been formulated with MPL to create an alternative to the AS04 adjuvant (30).

### 2.6 *No pain = no gain*? Unlinking adjuvanticity and reactogenicity

Reactogenicity has traditionally been viewed as being directly linked to increased immunogenicity of a vaccine and an inevitable aspect of inducing a strong immune response, even though there is limited data to support this concept (31). Indeed, certain aspects of the response are not required for robust adaptive immune induction (termed “wasted inflammation” by N.M. Valiante). Moreover, strong inflammatory responses can blunt T cell activation. While IL-1 is critically involved in the creation of a memory T cell pool, excessive inflammation negatively impacts memory cell expansion (32–34). This has significant practical implications since the extent and duration of a vaccine-induced inflammatory response affects the optimal interval between boosts, with non-inflammatory adjuvants/vaccine formulations having the potential to significantly shorten the immunization regimen without a negative impact on immunogenicity. Different strategies have been employed to uncouple reactogenicity from adjuvanticity: one approach is the “detoxification” of naturally occurring immunostimulators, as pioneered for LPS to create MPL (in this case, “detoxification” refers to reduction in excessive inflammation, which is different than the removal of the enterotoxic activity of LT in the case of the protein adjuvant dmLT^1^ (35). In this case, toxicity that is not associated with immunogenicity was removed through targeted mutations). In a more targeted manner, synthetic MPL-derivatives have been created that change the balance of the MyD88 *vs*. TRIF signaling through TLR4, thus reducing the inflammatory component of the signal while maintaining strong adjuvanticity. A similar approach was applied to the saponin QS21, a potent adjuvant that requires extensive formulation to address its reactogenicity. Systematic structure-activity-relationship studies have identified components of the molecule that are required for adjuvanticity as well as components that are responsible for reactogenicity and hemolytic activity, resulting in the design of the semi-synthetic derivative Titerquil-1055 in which the two undesirable features have been removed while retaining adjuvant activity. Next-generation saponin adjuvants, such as VSA-1^11^ and VSA-2^12^, are building upon these insights and further increase the attractiveness of saponin adjuvants.

## 3 *Quo vadis, adjuvants?* Re-defining what is an adjuvant

**Figure 2:**
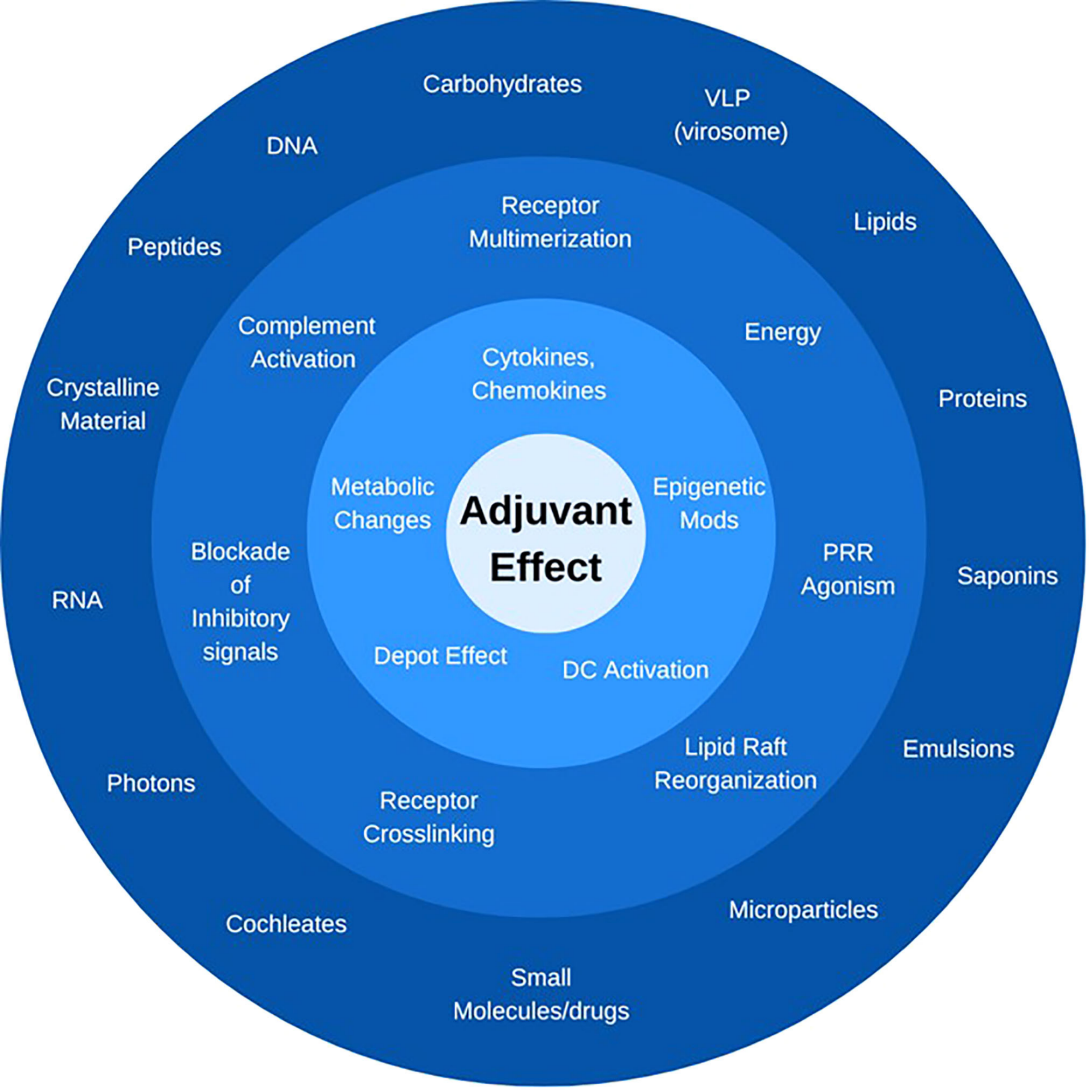
*What is an adjuvant?* Immunostimulators can be defined by their chemical nature and structure (*outer ring*), their activity and receptor through which they signal (*middle ring*), or the downstream cellular and molecular effect they trigger (*inner ring*). The only unifying feature is the ultimate “adjuvant effect”, i.*e.*, the enhancement of adaptive immune responses to a co-delivered antigen. In each category, only representative compounds or signals are listed.

The FDA’s definition of adjuvants is based on the outcome of the response they induce, *i.e.*, the enhancement of adaptive immune responses, not the mechanism by which this is achieved or even the nature of the immunostimulator. As a result, this definition captures a wide range of classes of molecules (*e.g*., proteins, lipids, carbohydrates, small molecules, nucleic acids) and formulations (*e.g*., emulsions, particulate delivery systems). Furthermore, it also includes non-physical types of immunostimulators, such as different forms of energy (laser light or specific radiofrequencies that activate innate immune cells) (**Figure 2**). An exciting and highly promising approach to achieve the desired adaptive immune response while controlling undesirable signals and “wasted inflammation” is the addition of “adjuvant modulators”. These are co-adjuvants that may or may not have adjuvant activity themselves. They modulate the signal(s) triggered by a ‘traditional’ PRR agonist through blocking of inflammatory signals while still preserving the adjuvant properties of the primary immunostimulator. The addition of partial NF-κB inhibitors to TLR agonists was shown to almost completely suppress the inflammatory response normally induced by the TLR agonist, while increasing, rather than reducing, the antibody response to a co-delivered antigen (36). Within this category of novel co-adjuvants are well-established compounds such as honokiol that partially inhibits the phosphorylation of the NF-κB p65 subunit (37), and capsaicin. The latter also has the potential benefit of being an analgesic which may further mitigate injection site pain. A variety of novel, selective inhibitors of NF-κB and other signaling pathways are currently in the development pipeline and have the potential to revolutionize the adjuvant field. While no vaccine with a synthetic signal inhibitor has been approved yet, the presence of structural variants of the molecule that act as partial agonists in MPL manufactured from Salmonella-endotoxin (LPS) was shown to be an additional mechanism responsible for the excellent safety profile of this adjuvant (38). To confirm the function of the antagonists naturally present in MPL preparations, Wang et al. used synthetic TLR4-antagonists to modulate the activity of synthetic TLR4-agonists. Through this proof-of-concept study (38), they also established how a *direct* antagonist of the pathway that an adjuvant induces can be employed to control and direct adjuvanticity without the need to structurally modify an adjuvant to tweak its activity, representing an alternative to the above-described addition of selective signaling pathway antagonists used as co-adjuvants.

### 3.1 (Re)-defining what is an adjuvant: The role of “*carriers*” such as lipid nanoparticles

Countless attempts have been made to categorize adjuvants and a frequently used, high-level categorization puts adjuvants either into the silo of “immunopotentiators” or “delivery vehicles”. While this approach implies, incorrectly, that a vaccine delivery system cannot also be an immunopotentiator, it nevertheless acknowledges that delivery vehicles may also provide an adjuvant effect. The field of either immunologically silent or immunostimulatory vaccine delivery systems is large and complex (39), and goes far beyond the scope of this article. It includes – to name just a few - virosomes (reviewed in (40)) that are among the few adjuvants in a licensed vaccine (41), a wide variety of liposomes, polymers such as carbomer-lecithin adjuvant Adjuplex (42) and PLGA nanoparticles that have been used to deliver various payloads (such as immunostimulatory vaccines for cancer (43) or tolerogenic vaccines for the treatment of autoimmune disease (44), and even immunologically inert materials such as gold particles for the biolistic delivery of DNA vaccines by gene gun (45) (which exert their adjuvant effect through the cellular trauma caused by high-velocity microparticle bombardment of the skin). A delivery system that has recently received unprecedented attention due to the COVID pandemic is lipid nanoparticles (LNPs) that are used in the approved mRNA vaccines BNT162b2/Comirnaty and mRNA-1273/Spikevax. LNPs are sub-micron particles that contain ionizable cationic lipids, as well as other types of lipids (reviewed in (46)). Interestingly, most of the recent attention has focused on the role of LNPs as delivery vehicles that protect the sensitive mRNA cargo from otherwise rapid degradation. In addition to various types of nucleic acids (including siRNA, microRNA, DNA), subunit-based vaccines have been delivered in various LNP formulations, to achieve different types of outcomes such as inducing immune responses or for gene therapy.

In an LNP-formulated mRNA vaccine, what provides the adjuvant effect? While studies with mRNA-based vaccines have been ongoing for at least three decades, their further development had been hampered by poor immunogenicity. This shortcoming was initially blamed on two proposed mechanisms: rapid degradation of mRNA by RNases *in vivo* (resulting in insufficient antigen expression) and the lack of endogenous adjuvanticity. In contrast, self-replicating RNA (srRNA, replicon RNA, or self-amplifying RNA (SAM), appeared to overcome both of these shortcomings by triggering high levels of antigen expression in transfected cells, while the process of RNA replication itself was shown to provide a potent adjuvant signal by activating different innate immune sensors (47–49). However, the significant difference in the efficacy provided by the LNP-formulated COVID mRNA vaccines BNT162b2 and mRNA-1273 compared to CVnCoV (developed by CureVac N.V. and CEPI) highlighted the impact of the immunostimulatory activity of mRNA. A key difference between the first two and the latter vaccine is that only CVnCoV employed non-modified mRNA, while the other two were based on modified mRNA in which uridine is replaced with pseudouridine (Ψ). This not only improves stability but also reduces – or even eliminates – innate immune stimulation by the mRNA construct (50). mRNA vaccines were shown to activate TLR3 (51) as well as TLR7 when specific sequence motifs (*i.e*., several uridines in close proximity) are present and the mRNA gains access to the endosomal compartment (52). While beneficial in the context of a subunit vaccine, the activation of innate immune pathways interferes with protein translation and, thus, protein production from the mRNA vaccine. In a modified, immunologically silent, mRNA vaccine, adjuvanticity is, therefore, provided mostly – or even exclusively, depending on the extent of the modifications – by the LNP formulation. Immune stimulation by LNPs depends on factors such as the nanoparticles’ size, shape, and rigidity, in addition to their composition. A key component of LNPs responsible for their adjuvant activity is ionizable lipids (53), however, the immunological mechanism by which LNPs provide their adjuvant effect are still under investigation. While the adjuvanticity of the DOTAP-containing LNP formulation from Acuitas Therapeutics was reported to be independent of MyD88 and MAVS thus suggesting no sensing by innate immune receptors (53), the response induced by particles based on another cationic lipid, lipopolyamine, appear to involve canonical innate immune receptors such as TLR2 (54). In this context, it should be noted that TLR2, together with TLR4, were reported to also be triggered by another nanoparticulate adjuvant/delivery system (55), the oil-in-water Nanoemulsion system W805EC (Nanovax, NE01)^13^ in the absence of receptor binding.

Immune stimulation by LNPs represents both challenge and opportunity: strong inflammatory responses triggered by LNPs can still interfere with antigen expression and, thus, negatively impact immunogenicity. When cholesterol in an LNP formulation was partially replaced with the anti-inflammatory corticosteroid dexamethasone, TNFα induction was abrogated resulting in increased antigen expression from co-delivered mRNA (56). This conceptually straight-forward and elegant approach serves as a reminder that the adjuvanticity of LNPs is tunable through the incorporation of exogenous (non-inflammatory) immunostimulators, or selective immunosuppressors such as small-molecule inhibitors of pro-inflammatory signaling pathways as already described for TLR agonists above (36).

## 4 Adjuvant development: The art of walking the razor’s edge

Developing a vaccine with a novel adjuvant is a perilous journey where many promising immunostimulators are passed over in favor of adjuvants that are already used in late-stage or licensed vaccines. From a vaccine developer’s perspective, considering the costs and investment of time that goes into the preclinical development and clinical testing of a new vaccine, the risk of proceeding with a novel adjuvant may appear to outweigh the advantage of selecting an adjuvant with a strong clinical track record. Thus, the choice of adjuvant for a new vaccine is most commonly a business decision, and not based on parameters such as optimal compatibility of the adjuvant with the vaccine formulation or the type of immune response that the adjuvant would elicit. While aluminum-based adjuvants have an unparalleled track record and an impressive safety profile, it is important to remember that the safety profile of a vaccine is not determined by the adjuvant, but rather the combination of adjuvant and vaccine antigen as well as formulation. The use of alum in an RSV-vaccine contributed to enhanced respiratory disease after infection (57). Even though obvious safety signals associated with a new adjuvant-antigen formulation would likely be detected in Phase I clinical trials, vaccine developers rightfully worry about rare and unforeseeable complications that may not show up until large Phase III trials have been completed, or – worse – until after the rollout of a vaccine. The identification of several cases of Bell’s Palsy after the introduction of an intranasal influenza vaccine adjuvanted with a novel bacterial adjuvant (heat-labile *E. coli* toxin) in Switzerland more than two decades ago (58) still reverberates through the vaccine community and is likely the primary reason for why so few intranasal vaccines using adjuvants are in the pipeline. But again, no adjuvant is immune from such an unfortunate outcome: AS03 (59) is a well-established, thoroughly tested, and “clinically de-risked” adjuvant used in pandemic influenza vaccines (Pandemrix and Arepanrix H1N1) that were deployed to more than 47 countries. Only after the vaccines’ widespread use, a small but measurable increase in cases of narcolepsy was noted, and only in select geographic areas (60). While infection with the H1N1 virus itself has been associated with breaking tolerance to a cross-reactive epitope on hypocretin (as well as narcolepsy-like sleep disruptions that do not appear to involve adaptive immune responses (61)), the combination of a strong adjuvant and a specific manufacturing process of the flu antigen, paired with the expression of specific HLA haplotypes triggered the autoimmune condition in an unforeseeable manner. This experience serves as a reminder that while reactogenicity may - to a large degree - depend on the nature and formulation of an adjuvant, overall vaccine safety is determined by additional factors such as the antigen the adjuvant is paired with, manufacturing, or the target population of the vaccine. Since adjuvants that had previously been used in humans do not come with a safety guarantee for any vaccine antigen or formulation, the selection of an adjuvant for a new vaccine should primarily be based on which type of immunostimulator promotes the most efficacious immune response, as determined in side-by-side comparison studies, regardless of what stage of development the adjuvant is at. Relying on aluminum-based adjuvants as the first - and often only - choice continues to slow the process of developing efficacious vaccines. Limited access to novel adjuvants should no longer be a limiting factor, with the large number of novel unique compounds and formulations that have been developed, and resources such as the NIAID Vaccine Adjuvant Compendium^14^ that facilitates access to novel adjuvants.

## 5 Conclusions

Adjuvant research and development has made enormous progress in recent years, which was further accelerated in an unprecedented manner by the SARS-CoV-2 pandemic. The global rush to address the pandemic with novel vaccines has allowed novel adjuvants to prove their usefulness, as well as their safety, in an unusually public fashion. Using a TLR7/8 agonist in hundreds of millions of vaccine recipients has shown how powerful innate immune stimulators can be tamed through appropriate adjuvant design or formulation. The approval of several COVID vaccines with novel adjuvants (Alhydroxiquim-II^9^, Advax/CpG55.2^8^, Matrix M, and Alum-CpG 1018) globally, and the preclinical and clinical development of countless more experimental COVID vaccines with novel adjuvants suggest that Rino Rappuoli’s observation several years ago that “Adjuvant development is one of the slowest processes in the history of medicine” (62) no longer applies. However, these recent successes should not distract from the various barriers that continue to shut many promising adjuvants out of the clinical development process. While the approval of COVID vaccines with novel adjuvants have created a demand for those specific adjuvants, vaccine developers continue to favor “clinically de-risked” adjuvants rather than being the first to take a vaccine with a novel adjuvant into the clinic. A second, scientific challenge that was brought to the forefront by the success of mRNA-based vaccines is the lack of an understanding of which adjuvants are useful – and even suitable – for nucleic acid-based vaccines. The induction of antiviral pathways intended to block viral replication cause many conventional adjuvants to interfere with antigen expression from nucleic acid vaccines thus quantitatively, as well as qualitatively altering the immune response. While the paucity of data from adjuvanted mRNA vaccines is a barrier for the development of improved mRNA formulations in the short run, it also represents an exciting research opportunity, and considering the strong interest in the mRNA vaccine platform, this information is urgently needed.

## Author contributions

All authors listed have made a substantial, direct, and intellectual contribution to the work and approved it for publication.

## Footnotes

1 VO_0005329; https://vac.niaid.nih.gov/view?id=13

2
https://vac.niaid.nih.gov/view?id=66

3
https://vac.niaid.nih.gov/view?id=29

4
https://vac.niaid.nih.gov/view?id=7

5
https://www.niaid.nih.gov/research/adjuvant-comparison

6
https://vac.niaid.nih.gov/view?id=7

7 VO_0005207; https://vac.niaid.nih.gov/view?id=37

8 VO_0005324; https://vac.niaid.nih.gov/view?id=38

9 VO_0005301; https://vac.niaid.nih.gov/view?id=12

10 VO_0005419; https://vac.niaid.nih.gov/view?id=25

11 VO_0005326; https://vac.niaid.nih.gov/view?id=41

12 VO_0005325; https://vac.niaid.nih.gov/view?id=42

13
https://vac.niaid.nih.gov/view?id=22

14
https://vac.niaid.nih.gov/
